# Antibiotic Resistances in Livestock: A Comparative Approach to Identify an Appropriate Regression Model for Count Data

**DOI:** 10.3389/fvets.2017.00071

**Published:** 2017-05-31

**Authors:** Anke Hüls, Cornelia Frömke, Katja Ickstadt, Katja Hille, Johanna Hering, Christiane von Münchhausen, Maria Hartmann, Lothar Kreienbrock

**Affiliations:** ^1^Faculty of Statistics, TU Dortmund University, Dortmund, Germany; ^2^IUF-Leibniz Research Institute for Environmental Medicine, Düsseldorf, Germany; ^3^Department of Biometry, Epidemiology and Information Processing, University for Veterinary Medicine Hanover, WHO-CC for Health at the Human-Animal-Environment Interface, Hannover, Germany

**Keywords:** veterinary epidemiology, model selection, overdispersion, underdispersion, zero inflation, Poisson regression, hurdle model

## Abstract

Antimicrobial resistance in livestock is a matter of general concern. To develop hygiene measures and methods for resistance prevention and control, epidemiological studies on a population level are needed to detect factors associated with antimicrobial resistance in livestock holdings. In general, regression models are used to describe these relationships between environmental factors and resistance outcome. Besides the study design, the correlation structures of the different outcomes of antibiotic resistance and structural zero measurements on the resistance outcome as well as on the exposure side are challenges for the epidemiological model building process. The use of appropriate regression models that acknowledge these complexities is essential to assure valid epidemiological interpretations. The aims of this paper are (i) to explain the model building process comparing several competing models for count data (negative binomial model, quasi-Poisson model, zero-inflated model, and hurdle model) and (ii) to compare these models using data from a cross-sectional study on antibiotic resistance in animal husbandry. These goals are essential to evaluate which model is most suitable to identify potential prevention measures. The dataset used as an example in our analyses was generated initially to study the prevalence and associated factors for the appearance of cefotaxime-resistant *Escherichia coli* in 48 German fattening pig farms. For each farm, the outcome was the count of samples with resistant bacteria. There was almost no overdispersion and only moderate evidence of excess zeros in the data. Our analyses show that it is essential to evaluate regression models in studies analyzing the relationship between environmental factors and antibiotic resistances in livestock. After model comparison based on evaluation of model predictions, Akaike information criterion, and Pearson residuals, here the hurdle model was judged to be the most appropriate model.

## Introduction

Antimicrobial resistance in livestock is a matter of public concern. In general, its occurrence is promoted by the exposure with antimicrobial substances and the subsequent selection of resistant bacteria as well as by the horizontal or vertical transfer of resistance determinants. To identify points of action for measures to prevent, reduce, and control antimicrobial resistance in farming and veterinary practice, epidemiological studies on a population level are crucial. These epidemiological studies usually lead to methodological constraints concerning sample size, zero measurements on the outcome as well as on the exposure side or associations between potential risk factors. Therefore, the study design as well as the characteristics of the collected data should be considered in the process of epidemiological model selection.

The diagnostic methods to identify antibiotic resistance as well as the statistical regression techniques that could be applied are manifold (e.g., Poisson model, negative binomial model, quasi-Poisson model, zero-inflated model, and hurdle model). To decide on the most suitable statistical model, a structured process of model selection is crucial to avoid misleading results and interpretation.

One major problem in analyzing bacterial data are structural zero measurements. Recently, two studies gave an overview about the impact of the choice of different probability distributions in the context of quantitative microbiological risk assessment to estimate the true prevalence and concentration of microorganisms in foods (1, 2). Duarte et al. (1) pointed out that when fitting a distribution to enumeration data from a sample of food products, several factors have an influence on the accuracy of the fit. First, enumeration of low contaminated sample units can give zero counts (“artificial” zeros) that add to the number of “true” zeros resulting from non-contaminated units, thereby inflating the total number of zeros in a sample of microbial counts. This results in a typically zero-inflated distribution of counts. They concluded that one of the keys to an accurate characterization of the overall microbial contamination was the correct identification and separation of true and artificial zeros (1).

To illustrate such a modeling process with the aim to identify potential factors associated with antibiotic resistance in livestock, data from a cross-sectional investigation on fattening pig farms in Germany were analyzed. In this cross-sectional study, 48 fattening pig farms in Germany formed the study population. In each farm, 10 samples were taken and tested for resistance against cefotaxime in *Escherichia coli* (*E. coli*) isolates. Information on the farm and the management of the animals were recorded with a questionnaire. The outcome used for the analyses was the count of positive samples [for more details, see Ref. (3)]. In the original publication of Hering et al. (3, 4), a generalized linear model (GLM) with logit link, which is the most popular approach for count data, was used.

The aims of this paper are to explain the model building process comparing competing models for count data with a special emphasis on the modeling of zero measurements and to compare these models using the study data as an example. Based on this comparison, we recommend a scheme how to evaluate statistical models for studies of antibiotic resistance in livestock.

## Materials and Methods

### Sample Data Set: Study Design

The cross-sectional investigation was part of the RESET research network.^1^ For this epidemiological study, representative districts were identified on the basis of average farm and animal numbers (4). The farmers were contacted at information sessions organized in cooperation with veterinary offices or agricultural associations in each district. Based on an approximate two-sided 95%-confidence interval for sample proportion, it was estimated that 50 farms were necessary to estimate a prevalence of cefotaxime resistance of 10–20% with an absolute error of ±10%. Overall, 48 farms with fattening pigs were visited between May 2011 and October 2012. In each farm, 10 samples were collected: six mixed fecal samples from the ground, two pairs of autoclaved boot swabs from the corridor inside the compartment, and two dust samples from the windowsill, pen separation, or automatic feeder. To assess factors associated with cefotaxime-resistant *E. coli*, a questionnaire dealing with different aspects of farm and animal management was applied. All microbiological analyses were carried out in the Institute for Animal Hygiene and Environmental Health of the Free University Berlin. A sample was defined as positive, if bacterial growth was observed on MacConkey agar containing 1 μg/ml cefotaxime and at least one colony could be confirmed as *E. coli* by Matrix-Assisted Laser Desorption Ionization TOF. A detailed description of the study design has been published previously (3).

### Sample Data Set: Description of Laboratory Results

Overall, 288 fecal samples, 96 pairs of boot swab samples, and 95 dust samples were collected. The proportion of positive samples was 61% for fecal samples, 54% for boot swabs, and 10% for dust samples. In seven farms, no cefotaxime-resistant *E. coli* were detected. On the remaining 41 farms (85%), at least one positive sample was observed.

### Sample Data Set: Basic Variable Selection

While the questionnaire consisted of 242 items that could potentially influence the outcome of resistance, in general, the management and structure of the observed pig farms was rather homogeneous. To avoid sparse data problems, 145 items were excluded from the analyses because less than five farms fell into one of the answer categories. Prior to multivariable regression analyses, a univariable analysis was performed for each of the remaining 95 items. Variables with a *p*-value smaller than 0.2 were included into the multivariable analyses to pre-select possible risk factors. Associations among predictor variables were investigated using Cramer’s V and also re-examined using the correlation matrix of the predictors from the multivariable model. Upon the univariable screening of variables and elimination of highly collinear factors, the final multivariable model contained 17 variables (3).

### Modeling Count Data: General Remarks

The standard method for analyzing count data is Poisson regression modeling (5). However, Poisson regression can often be inappropriate in practice due to over- or underdispersion, i.e., the variance of the data does not fit the general assumption of the Poisson distribution, that the expected mean μ and variance σ^2^ of counts are the same. This usually is measured with the dispersion index ϕ = σ^2^/μ. In addition, an excess number of zeros is often observed, which could not be modeled by the Poisson distribution.

To overcome these pitfalls of the Poisson model, two common options to handle overdispersion are the negative binomial model and the quasi-Poisson model (6). Methods to handle zero inflation include zero-inflated models or hurdle models (6). Several applications and comparisons of Poisson, negative binomial, zero-inflated, and hurdle models have been published in the literature [for example, Ref. (7–10)]. The statistical background of these models will be summarized in the following subsection. For more details, please see Ref. (11).

### Modeling Count Data in the Presence of Overdispersion and Zero Inflation

In epidemiology, the Poisson distribution is the most common model for the analysis of count data. Assuming a cross-sectional study design, let *y_i_* denote the count of positive samples on the *i*th farm (*i* = 1,…, *n*) and let *x_i_* denote a (*k* + 1)-dimensional set of corresponding factors on the *i*th farm. Following a Poisson distribution

(1) $$f_{\text{poisson}}\left(y;\mu \right)=\frac{\text{exp}\left(-\mu \right)\cdot \mu^{y}}{y!}$$

with mean μ the random variables *Y_i_*|*X_i_* = *x_i_* in the Poisson model have mean and variance *E*(*Y_i_*|*X_i_*) = Var(*Y_i_*|*X_i_*) = μ*_i_* which leads to a dispersion index of ϕ = 1 (5, 12, 13). The Poisson model belongs to the class of GLMs (13, 14). The corresponding regression equation for the mean is

$$\mu_{i}=\text{exp}\;\left(x_{i}^{T}\beta \right)\;\;,$$

with the parameter vector β = (β_1_,…, β*_k_*) representing the effect of each of the *k* factors.

**Figure 1:**
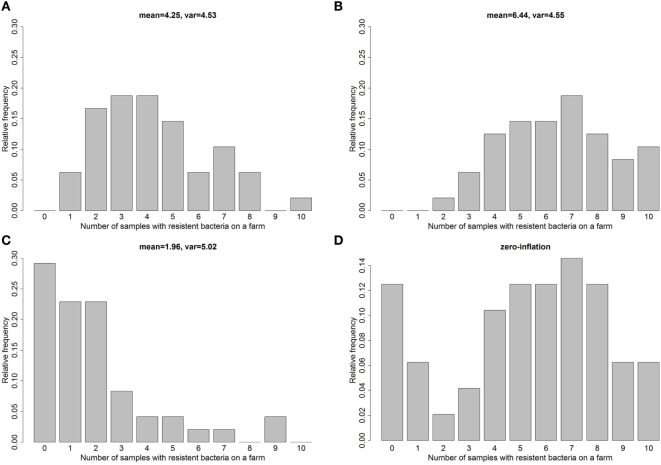
**The histogram illustrates simulated count data from a Poisson distribution (A), with underdispersion (B), and with overdispersion (C) and count data with zero inflation (D) motivated by the real data example for antibiotic resistances in 48 German fattening pig farms**.

In real data sets, the assumption that the dispersion ϕ = 1 is often violated. The consequence of an underdispersion [ϕ < 1, i.e., Var(*Y_i_*|*X_i_*) < *E*(*Y_i_*|*X_i_*)] is that SEs are too conservative. In models with an overdispersion [ϕ > 1, i.e., Var(*Y_i_*|*X_i_*) > *E*(*Y_i_*|*X_i_*)], which is more common, the level of significance is not kept. As an example, Figure 1 shows the distribution of count data simulated from a regular Poisson distribution (Figure 1A), count data with underdispersion (Figure 1B) and count data with overdispersion (Figure 1C). Therefore, the first step in analyzing count data is to identify over- or underdispersion of the fitted Poisson model.

One way to account for overdispersion in the regression model is to assume a negative binomial distribution for the outcome *Y_i_*|*X_i_*, which formally can be described as a gamma mixture of Poisson distributions (6). The probability density function is defined as

(2) $$f_{\text{negbin}}\left(y;\mu ,\theta \right)=\frac{\Gamma \left(y+\theta \right)}{\Gamma \left(\theta \right)\cdot y!}\cdot \frac{\mu^{y}\cdot \theta^{\theta }}{{\left(\mu +\theta \right)}^{y+\theta }},$$

with mean μ and shape parameter θ; Γ(⋅) is the gamma function. Again the corresponding regression equation for the mean is

$$\mu_{i}=\text{exp}\;\left(x_{i}^{T}\beta \right).$$

Another way to handle moderate overdispersion (or underdispersion) is the quasi-Poisson regression model, which introduces the dispersion index ϕ in the Poisson model, so that the variance of the response is modeled as a linear function of the mean:

$$\text{Var}\;\left(Y_{i}|X_{i}\right)=\phi \mu_{i}.$$

The dispersion index ϕ can be estimated by

$$\hat{\phi }=\frac{1}{n-k}\;\sum \frac{{\left(y_{i}-{\hat{\mu }}_{i}\right)}^{2}}{{\hat{\mu }}_{i}},$$

with *n* observations, *k* risk factors, and using ${\hat{\mu }}_{i}$ as a so-called plug-in estimate for μ*_i_* (6, 15).

A second problem with count data can be an excessive number of zero counts (zero inflation) that cannot be suitably modeled by the Poisson or negative binomial distribution. This problem often appears in resistance research, where a lot of samples are negative [e.g., in broiler chicken, see Ref. (16, 17)] or *vice versa* [e.g., Ref. (18)]. An excess of zeros can be easily identified through a histogram of the observed count data. Figure 1D shows an example of simulated zero-inflated count data.

In the presence of a large proportion of zero counts, a zero-inflated model or hurdle model should be considered as alternatives to the Poisson or the negative binomial models (6, 8, 10).

The idea of the zero-inflated model is that some of the zeros are modeled to be part of the Poisson (or negative binomial) distribution, while the other part of the zeros are modeled through a binomial distribution (8). In our study, zero-inflated model results may be interpreted as part of the farms having no resistant bacteria at all, and in contrast, other farms, where the risk of resistant bacteria has been eliminated actually. Indeed, this point of view assumes that the study design is free from any misclassification. If false-negative results appear, the above interpretation is biased by adding false-negatives to the number of “true” zeros, which was described by Ref. (2).

Unlike zero-inflated models, hurdle models do not make the distinction between different types of zeros and handle them identically (19). Therefore, these models include all three type of zeros by definition. The basic idea is that a binomial probability model governs the binary outcome of whether a count variable is zero or positive. If the realization is positive, the “hurdle” is crossed, and the conditional distribution of the positives is governed by a truncated at-zero count data model (20). In the study addressed here, all zeros in a hurdle model represent farms without any resistant bacteria (true negatives and false-negative results), whereas the count-part of the model describes the situation of farms that did exhibit resistant bacteria.

To distinguish between zero-inflated/hurdle models with a Poisson and those with a negative binomial, the overdispersion within the count-part of the data needs to be evaluated. One option might be to evaluate the dispersion index after excluding all zero counts. However, since we cannot determine which of the zeros belong to the count-part and which belong to the zero-part, the dispersion index might be biased toward underdispersion after excluding all zeros. For a clarification, the histogram of the data distribution needs to be evaluated and compared to simulated data with overdispersion (compare Figure 1) and if there is some indication for overdispersion, Akaike information criterion (AIC), and Pearson residuals of the zero-inflated/hurdle models with negative binomial distribution should be compared to zero-inflated/hurdle models with Poisson distribution.

In more formal detail, zero-inflated models (20, 21) are two-component mixture models that combine a point distribution *I*_{0}_(*y*) (e.g., binomial) with a count distribution *f*_count_(*y*; *x*, β) (Poisson or negative binomial). This leads to a two-component probability density function defined as

$$f_{\text{zeroinfl}}\left(y;x,z,\beta ,\gamma \right)=f_{\text{zero}}\left(0;z,\gamma \right)\cdot I_{\left\{0\right\}}\left(y\right)+\left(1-f_{\text{zero}}\left(0;z,\gamma \right)\right)\cdot f_{\text{count}}\left(y;x,\beta \right).$$

The parameter vectors β and γ can be estimated using maximum likelihood. The corresponding regression equation for the mean is given by

$$\mu_{i}=f_{\text{zero}}\left(0;z_{i},\gamma \right)\cdot 0+\left(1-f_{\text{zero}}\left(0;z_{i},\gamma \right)\right)\cdot \text{exp}\left(x_{i}^{T}\beta \right)\;\;.$$

In contrast, the probability density function of the hurdle model is defined as

$$f_{\text{hurdle}}\left(y;x,z,\beta ,\gamma \right)=\left\{\begin{array}{ll} f_{\text{zero}}\left(0;z,\gamma \right)\; & \;\text{if}\;y=0\\ \left(1-f_{\text{zero}}\left(0;z,\gamma \right)\right)\cdot f_{\text{count}}\left(y;x,\beta \right)∕\left(1-f_{\text{count}}\left(0;x,\beta \right)\right)\; & \;\text{if}\;y>0 \end{array}\right.$$

with a count data model *f*_count_(*y*; *x*, β), such as the Poisson or negative binomial distribution (Eqs 1 and 2), that is left-truncated at *Y* = 1 and a zero hurdle model *f*_zero_(*y*; *z*, γ) that is right-censored at *Y* = 1 (6). The parameter vectors β and γ can be estimated through maximum likelihood. The corresponding regression equation for the mean is given by

$$\log\left(\mu_{i}\right)=x_{i}^{T}\beta +\log\left(1-f_{\text{zero}}\left(0;z_{i},\gamma \right)\right)-\log\left(1-f_{\text{count}}\left(0;x_{i},\beta \right)\right).$$

Therefore, in general, zero-inflated Poisson (ZIP), zero-inflated binomial, and hurdle models are often much more appropriate to take into account the several ways of transmit resistance into farms.

### Sample Data Set: Variable Selection for Comparing Poisson Regressions

To decide which model fits best to our data, we first compared the observed counts with the model predictions, followed by a comparison of the AIC. The AIC is a measure of the goodness of fit of the model, which describes the trade-off between accuracy and complexity of the model. The model with minimum AIC value is preferred. Furthermore, we calculated the Pearson residuals and plotted them against the fitted values. If the model assumptions are suitable, the residuals should randomly fall within an area representing a horizontal band. Finally, we checked the plausibility of the estimates and their SEs. Inflated estimates and SEs might give a further hint for violated model assumptions.

Given the available sample size, we applied the following procedure to reduce the number of variables considered in the model comparison process. Using backward selection based on the AIC criterion, we retained eight variables in the Poisson model. Since the Poisson model discussed here is limited to 7 degrees of freedom only, the variables with the highest *p*-value after backward selection was excluded as well. The remaining variables were used for the count models following: Poisson, quasi-Poisson, and negative binomial; and for the count-part of the zero-inflated and hurdle models (Table 1). Additionally, on the basis of these seven variables, a backward selection by the AIC criterion was performed on the zero-part of the hurdle model, leading to five variables in the zero-part of the hurdle and the ZIP models (Table 2).

**Table 1:** **Description of variables included in the count models (Poisson, quasi-Poisson, and negative binomial model) and in the count-part of the zero-inflated Poisson and the hurdle model**.

	Farms with fattening pigs (%)
*N*	48
Moving single pigs,^a^ *n* (%)	39 (81.25)
Seperate pen for diseased pigs,^a^ *n* (%)	32 (66.67)
Use of purchased feed only,^a^ *n* (%)	11 (22.92)
Water birds in 1 km radius of farm,^a^ *n* (%)	17 (35.42)
Disinfection of livestock trail	
Never,^b^ *n* (%)	8 (16.67)
After housing out, *n* (%)	33 (68.75)
Less frequent than after housing out, *n* (%)	7 (14.58)
Disinfection with chlorine,^a^ *n* (%)	8 (16.67)
Number of fattening pigs	
0 to ≤1,000,^b^ *n* (%)	20 (41.67)
>1,000 to ≤1,500, *n* (%)	14 (29.17)
>1,500, *n* (%)	14 (29.17)

*^a^“No” as reference category*.

*^b^Reference category*.

**Table 2:** Regression estimates*, SEs and *p*-values of the variables estimated in the Poisson, quasi-Poisson, negative binomial, zero-inflated Poisson (ZIP), and hurdle model.

		Poisson			Quasi-Poisson			Negative binomial			ZIP			Hurdle		
		exp($\hat{\beta }$)	SE	*p*-Value	exp($\hat{\beta }$)	SE	*p*-Value	exp($\hat{\beta }$)	SE	*p*-Value	exp($\hat{\beta }$)	SE	*p*-Value	exp($\hat{\beta }$)	SE	*p*-Value
Count model/Count-part	Intercept	0.82	0.34	0.548	0.82	0.42	0.632	0.46	0.45	0.095	2.18	0.73	0.290	2.61	0.41	**0.018**
	Moving single pigs	1.76	0.22	**0.010**	1.76	0.27	**0.045**	1.83	0.31	0.061	1.72	0.28	0.056	1.73	0.24	**0.023**
	Separate pen for diseased pigs	1.74	0.20	**0.005**	1.74	0.25	**0.030**	1.85	0.30	**0.047**	1.50	0.26	0.127	1.44	0.21	0.084
	Use of purchased feed only	0.56	0.19	**0.003**	0.56	0.24	**0.022**	0.49	0.29	**0.018**	0.64	0.24	0.072	0.65	0.21	**0.042**
	Water birds in 1 km radius of farm	1.24	0.16	0.177	1.24	0.20	0.285	1.44	0.26	0.170	1.10	0.17	0.576	1.09	0.16	0.592
	Disinfection of livestock trail after housing out	2.21	0.24	**0.001**	2.21	0.30	**0.011**	3.01	0.33	**0.002**	1.15	0.45	0.761	0.99	0.26	0.977
	Disinfection of livestock trail less frequent than after housing out	1.64	0.29	0.086	1.64	0.36	0.176	2.46	0.44	**0.046**	0.95	0.46	0.914	0.83	0.31	0.555
	Disinfection with chlorine	1.38	0.19	0.090	1.38	0.24	0.182	1.72	0.31	0.089	1.24	0.21	0.307	1.20	0.19	0.318
	Number of fattening pigs (>1,000 to ≤1,500)	1.07	0.17	0.697	1.07	0.22	0.756	1.19	0.29	0.547	0.93	0.18	0.696	0.93	0.18	0.700
	Number of fattening pigs (>1,500)	2.01	0.18	**1.10 × 10^−04^**	2.01	0.22	**0.004**	2.83	0.29	**0.001**	1.47	0.24	0.104	1.42	0.20	0.082

Zero-part^a^	Intercept										2.81	1.28	0.419	3.32	1.18	0.309
	Separate pen for diseased pigs										0.09	1.48	0.097	0.07	1.27	**0.037**
	Use of purchased feed only										6.32	2.16	0.393	5.04	1.33	0.223
	Water birds in 1 km radius of farm										0.10	2.6	0.382	0.16	1.55	0.240
	Disinfection with chlorine										0.00	7,210.56	0.998	0.00	5,847.27	0.998
	Number of fattening pigs (>1,000 to ≤1,500)										0.09	2.24	0.287	0.12	1.62	0.199
	Number of fattening pigs (>1,500)										0.02	3.86	0.331	0.04	1.66	0.056

	Akaike information criterion (AIC)	231.6385			231.6385			265.2818			233.2511			233.0947		

*The variables in the zero-part were chosen following a backward selection based on the AIC of the hurdle model*.

*Interpretation of estimates in the count model/count-part: a one unit change in the predictor variable is associated with a $\left(\text{1}-\text{exp}\left(\hat{\beta }\right)\right)*$100 percentage change of the expected sample count, holding other variables constant. Interpretation of estimates in the zero-part: a one unit change in the predictor variable is associated with a $\left(\text{1}-\text{exp}\left(\hat{\beta }\right)\right)*$100 percentage increase of the odds (chance) of not having any positive samples.

*^a^The ZIP model estimates the probability for zero inflation and the hurdle model estimates the probability for hurdle crossing (non-zeros). Therefore, the estimates of the hurdle model were multiplied by (−1) to make them comparable to the ZIP model*.

*Bold: p-values that passed the significance threshold (*p* < 0.05)*.

### Sample Data Set: Statistical Software

Analyses were performed using R 3.0.3 (22). The negative binomial model was fitted using the *glm.nb* function from the *MASS* package (23), the quasi-Poisson model with the *glm()* function by setting *family* = *quasipoisson()* (6), and the hurdle model and the zero-inflated models with the *hurdle()* and *zeroinfl()* functions, respectively, from the *pscl* package (6, 24).

## Results: Application to the Reset Study

**Figure 2:**
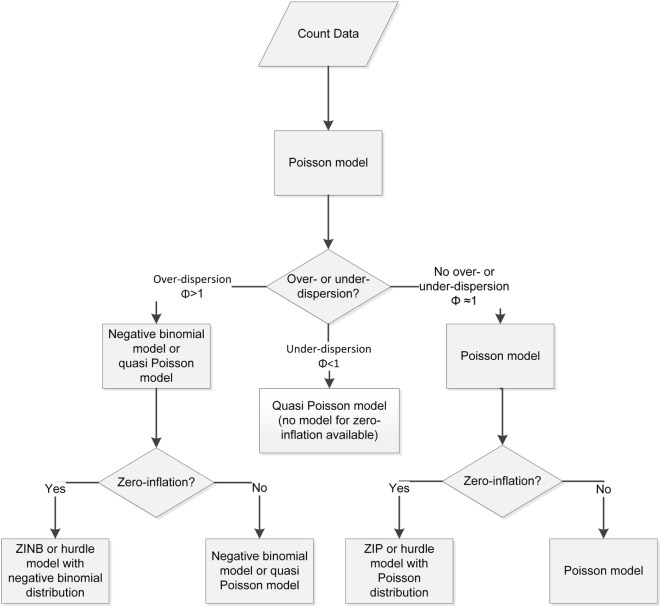
**Flow chart for model building processes for count data**.

Forty-eight fattening pig farms in Germany were investigated. For each farm, the primary outcome was the count of samples with cefotaxime-resistant *E. coli* (positive samples). To ensure a structured approach in evaluating the competing models, a model building strategy was established with a Poisson model as a starting point (Figure 2).

**Figure 3:**
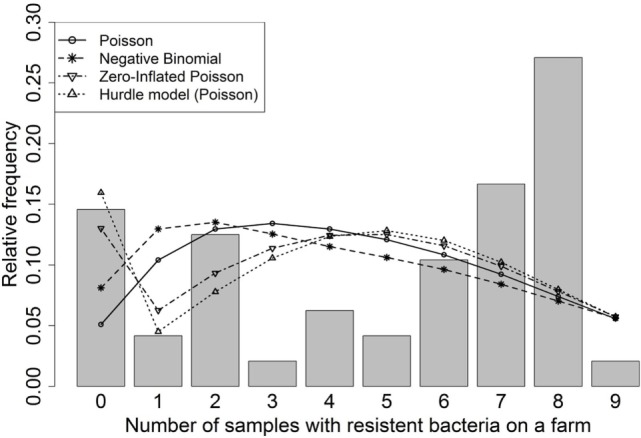
**The histogram illustrates the observed count data**. The lines show the predictions from the different regression models [Poisson, negative binomial, zero-inflated Poisson, hurdle model (Poisson)].

In our analysis, the Poisson model exhibited slight overdispersion (dispersion index ϕ = 1.55). Therefore, we investigated the negative binomial and quasi-Poisson models within the model selection process. The descriptive analysis of the observed data (Figure 3) indicated some zero inflation. In total, 14.6% of the samples were zero measurements potentially some being excess zeros and some being zeros from the counting data part (Poisson or NB), leading us to consider a hurdle model (with Poisson distribution) and the ZIP model. We did not consider zero-inflated/hurdle models with a negative binomial distribution for the count-part of the model as there did not appear to be an evidence of overdispersion in this setting after excluding the zero counts [ϕ = 1.02 and no descriptive indication for overdispersion in the count-part (compare simulated count data in Figure 1 and observed count data in Figure 3)].

Comparing the model predictions for the Poisson, negative binomial, ZIP, and hurdle models (Figure 3), the predicted outcomes for the regression parameters for four or more positive samples were all similar. For a smaller number of positive samples, the model predictions estimated in the ZIP and hurdle model were almost identical and fitted well the observed data, whereas the model predictions from the Poisson and negative binomial models exhibited a lack of fit. The model with the smallest AIC was the Poisson model, followed, in ascending order, by the hurdle, ZIP, and negative binomial models (Table 2).

**Figure 4:**
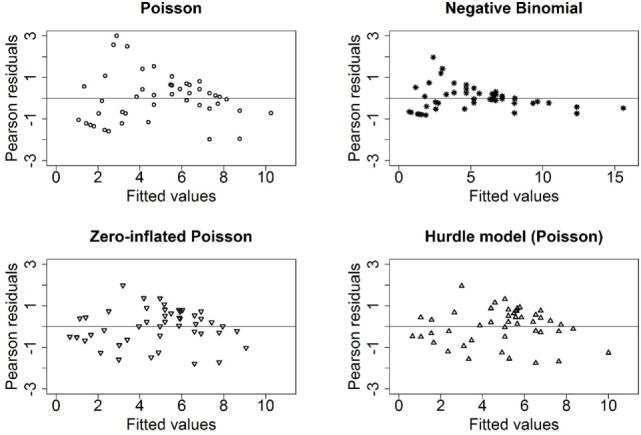
**Residual plots (Pearson residuals against the fitted values) of the four models for count data in our study**.

The Pearson residuals for the Poisson and the negative binomial models appeared to violate the assumption of homoscedasticity (Figure 4). Furthermore, the assumption of a larger variance in the negative binomial model led to a wider range of fitted values (0.7–15.6) indicating a poor model fit. The residuals for the zero-inflated models (ZIP, hurdle) did not indicate any issues with model fit. In summary, while the Poisson model had the smallest AIC, the model predictions, and Pearson residuals indicated the ZIP or hurdle model provided better model fit.

The model with the highest number of statistically significant factors associated with antibiotic resistance was the Poisson model (Table 2). The estimates for the Poisson and quasi-Poisson model are identical by definition, but the SEs and resulting *p*-values for the quasi-Poisson model were larger due to overdispersion. The count-part of the hurdle model indicated a statistically significant association between two variables (“moving single pigs” and “origin of feed”) and a higher number of positive samples, while the zero-part of the model showed a statistically significant association between “having a separate pen for diseased pigs” and a higher number of positive samples. However, in the ZIP model, there were no statistically significant effects due to larger SEs. Nevertheless, estimates for the variables of “moving single pigs” and “origin of feed” were similar to the hurdle model and the corresponding *p*-values were only slightly above 0.05.

Next, a variable selection was performed to decide which of these two models should be applied. In the main analysis, factors for the zero-part of the ZIP and hurdle model were chosen using a backward selection procedure based on the hurdle model resulting in the following factors included in the zero-part: “separate pen for diseased pigs,” “use of purchased feed,” “water birds in 1 km radius of farm,” “disinfection with chlorine,” and “number of fattening pigs” (Table 2). For the sensitivity analysis, the above analysis was repeated by choosing factors for the zero-part of these models using a backward selection procedure based on the ZIP model (Table 3). This resulted in retaining the three factors “use of purchased feed,” “water birds in 1 km radius of farm,” and “number of fattening pigs” instead of the five factors initially retained (Table 3). In this case, the AIC of the ZIP model was even smaller than that of the Poisson model. For the count-part of the ZIP model, four factors were statistically significant with effect estimates similar to that of the Poisson and hurdle models. However, the results for the zero-part of the ZIP model yielded inflated estimates and SEs for nearly each factor under study, whereas this was not the case for the hurdle model (Table 3).

**Table 3:** **Sensitivity analysis: the variables in the zero-part were chosen following a backward selection based on the Akaike information criterion (AIC) of the zero-inflated Poisson (ZIP) model instead of the hurdle model (compare Table 2)**.

		ZIP			Hurdle		
		exp($\hat{\beta }$)	SE	*p*-Value	exp($\hat{\beta }$)	SE	*p*-Value
Count-part	Intercept	1.54	0.38	0.264	2.61	0.41	**0.018**
	Moving single pigs	1.82	0.23	**0.008**	1.73	0.24	**0.023**
	Separate pen for diseased pigs	1.66	0.20	**0.011**	1.44	0.21	0.084
	Use of purchased feed only	0.62	0.20	**0.015**	0.65	0.21	**0.042**
	Water birds in 1 km radius of farm	1.10	0.16	0.537	1.09	0.16	0.592
	Disinfection of livestock trail after housing out	1.39	0.27	0.229	0.99	0.26	0.977
	Disinfection of livestock trail less frequent than after housing out	1.13	0.32	0.703	0.83	0.31	0.555
	Disinfection with chlorine	1.30	0.19	0.158	1.20	0.19	0.318
	Number of fattening pigs (>1,000 to ≤1,500)	0.94	0.18	0.712	0.93	0.18	0.700
	Number of fattening pigs (>1,500)	1.59	0.19	**0.018**	1.42	0.20	0.082

Zero-part^a^	Intercept	0.38	0.68	0.151	0.40	0.58	0.114
	Use of purchased feed only	>1,000	>1,000	0.993	3.63	1.14	0.259
	Water birds in 1 km radius of farm	0.00	>1,000	1.000	0.19	1.26	0.185
	Number of fattening pigs (>1,000 to ≤1,500)	0.00	>1,000	1.000	0.22	1.20	0.214
	Number of fattening pigs (>1,500)	0.00	>1,000	0.988	0.15	1.30	0.148

	**AIC**	229.91			236.71		

Estimates*, SEs, and *p*-values of the variables are given for the ZIP and hurdle model.

*Interpretation of estimates in the count model/count-part: a one unit change in the predictor variable is associated with a $\left(\text{1}-\text{exp}\left(\hat{\beta }\right)\right)*$ 100 percentage change of the expected sample count, holding other variables constant. Interpretation of estimates in the zero-part: a one unit change in the predictor variable is associated with a $\left(\text{1}-\text{exp}\left(\hat{\beta }\right)\right)*$ 100 percentage increase of the odds (chance) of not having any positive samples.

*^a^The ZIP model estimates the probability for zero inflation and the hurdle model estimates the probability for hurdle crossing (non-zeros). Therefore, the estimates of the hurdle model were multiplied by (−1) to make them comparable to the ZIP model*.

*Bold: p-values that passed the significance threshold (*p* < 0.05)*.

Therefore, for our dataset we conclude that the hurdle model is more stable than the ZIP model. Furthermore, interpretation of the zero-part of the hurdle model is much more in line with the underlying biology of the transmission of antibiotic resistance. Factors in the zero-part of the model may be related to farms without any resistant bacteria at all, whereas factors in the count-part of the model may be interpreted as factors associated with the success of handling the resistance problem.

In contrast to the results by Hering et al. (17), the factors associated with antibiotic consumption presented here are slightly different. Variables that were applied to backward selection both, in Hering et al. (17) and in the present publication were: “Separate pen for diseased pigs,” “Moving single pigs,” “Water birds in 1 km radius of farm,” “Disinfection of livestock trail,” and “Disinfection with chlorine.” The only factor with a *p*-value < 0.05 in both publications was “Separate pen for diseased pigs,” which is a measure of hygiene indeed. Additional factors with a *p*-value < 0.05 in the multiple Poisson regression by Hering et al. (17) were “Ventilation” and “Control of flies with toxin” while in the analyses presented here the variables “Moving single pigs,” “Use of purchased feed,” “Disinfection of livestock trail,” and “Number of fattening pigs (>1,500)” were identified also. In fact, the factors identified here, seem to be more in line with the biology of antibiotic resistance transmission. But, the results of the Poisson model published earlier cannot be compared directly with the results of the Poisson model presented here because the hierarchical level of sampled animal group within farm was not considered due to guarantee an “overall-stability” in the modeling process.

In summary, we conclude, therefore, that the hurdle model is the most appropriate model to analyze our data, despite the fact that the Poisson model achieved the lowest AIC. While the residual plot of the Poisson model indicated variance heterogeneity and partly larger residuals, the model predictions and Pearson residuals indicated the hurdle model provided better model fit with plausible estimates and SEs.

## Discussion and Recommendations

In this paper, we address the problem of statistical model building and selection for studies analyzing the association of antibiotic resistance with environmental factors. These regression models based on epidemiological data on a population level are needed to identify potential intervention measures to reduce the occurrence and transmission of resistance.

The Poisson model is the most common model for count data (here, number of resistant samples in a farm). However, in many situations, this is not an appropriate model due to violations of the general model assumptions. Therefore, it is essential to evaluate the model assumptions for the Poisson model in a systematic way in order to identify relevant factors associated with resistance. Hence, the main objective of the paper was to demonstrate possible strategies for model selection, taking into account the two most important problem areas when modeling count data, namely, over- or underdispersion and zero inflation, which applies especially on epidemiological studies on the occurrence of antimicrobial resistance in livestock.

Strong overdispersion can be handled through the use of a negative binomial model. For more subtle overdispersion and even underdispersion, the quasi-Poisson model can be used to correct the SEs and resulting *p*-values, while zero-inflated models or the hurdle model can be used to handle zero inflation. However, the application of all discussed models is restricted to the case of fixed sample size per herd. In the case of different sample sizes, the observations need to be transformed so that they can be modeled by the same distribution. Furthermore, we did not account for the different nature of samples in the sample set. This could have either been done by modeling a hierarchical binomial distribution instead of summarizing all samples to one count variable or by weighting the samples according to their nature.

In practical studies, over- or underdispersion and zero inflation are accompanied with the problem of misclassification of outcome. For the topic addressed here, observed zeros are “true zeros” and “false-negative zeros,” where within the “true zeros” farms were included with no resistance at all. As in other epidemiological studies in veterinary science, this aspect has to be discussed in detail, especially in the light of the multistep isolation procedure suggested in this study. In principal, this is due to two components, the laboratory sensitivity as well as the sampling sensitivity. In our study, the diagnostic protocol was developed with a special emphasis on identifying ESBL positives, i.e., with enrichment on the resistant bacteria. This protocol developed in the RESET-consort meanwhile is approved by the EFSA. In general, it is stated that the diagnostic sensitivity is close to 100% due to the enrichment procedures. Therefore, the impact of false-negatives may be neglected from the laboratory process and may appear only by the sampling procedure. Here, 10 samples per farm were chosen following the general concept of EFSA-baseline studies to narrow this error.^2^ Taking these both points into account, the number of false-zeros may be neglected in our study and models including the false-negative rate were not incorporated into the model building process.

This paper investigates statistical models for the analyses of count data using a study of cefotaxime-resistant *E. coli* in German fattening pig farms as an example for the model selection process. In general, results of epidemiological multifactor models vary depending on the choice of the statistical model. Therefore, it is not recommended to strictly define the models to be used in the study protocol but to define the model building process, depending on whether the final data fulfill the model assumptions. In detail, our results indicate that for analyzing count data of antibiotic resistance in small study populations (*n* = 48), the hurdle model might be more appropriate to handle a moderate excess of zeros which is in line with Xu et al. (19) who pointed out that the hurdle models are more stable when structural zeros are absent. However, given our application to only a single dataset, more comprehensive studies would be needed to confirm whether our findings can be generalized. Furthermore, if structural zeros are believed to exist due to misclassification and the interest is in modeling them, the zero-inflated models should be chosen. The probability to observe counts greater than zero in the Poisson or negative binomial part of the model can then be interpreted as diagnostic sensitivity. However, in this case, if non-convergence is encountered, a larger sample size is probably required (19). In addition, our results show that the decision for the most appropriate model should not be based on the AIC only. It is important to compare the observed values with the model predictions and to perform a residual analysis (Pearson residuals).

Motivated by our analysis, we conclude that the recommendation scheme provided in Figure 2 should be used to find the most appropriate method to handle count data in the case of over-/underdispersion and/or zero inflation.

## Author Contributions

Study design and data collection: JH, KH, CM, and LK. Data management and analyses: AH, CF, KI, MH, and LK. Wrote the paper: AH, CF, KI, KH, and LK.

## Conflict of Interest Statement

The authors declare that the research was conducted in the absence of any commercial or financial relationships that could be construed as a potential conflict of interest.

## Footnotes

^1^www.reset-verbund.de.

^2^https://www.efsa.europa.eu/.

## Abbreviations

*E. coli, Escherichia coli*; ZIP, zero-inflated Poisson; ZINB, zero-inflated negative binomial; AIC, Akaike information criterion.
